# Genome Sequence of Chinese Porcine Parvovirus Strain BJ2

**DOI:** 10.1128/mra.00094-23

**Published:** 2023-06-05

**Authors:** Wenying Zhao, Yujie Sun, Linghua Guo, Yunjing Zhang, Baicheng Huang, Kegong Tian

**Affiliations:** a National Research Center for Veterinary Medicine, Luoyang, China; Portland State University

## Abstract

Porcine parvovirus (PPV) strain BJ2 was isolated from a pig with symptoms consistent with PPV in Beijing, China. The analysis showed that the PPV genome sequence has the characteristics of a German cluster 27a strain and the virulent Kreese strain, which will facilitate understanding of the prevalence of PPV in China.

## ANNOUNCEMENT

Porcine parvovirus (PPV) is one of the main pathogens causing reproductive disorders in sows. The main clinical symptoms are sow infertility, abortion, mummified fetuses, and delayed estrus (1, 2). PPV is a nonenveloped, negative-stranded, single-stranded linear DNA virus and is a member of the genus *Protoparvovirus*. The PPV genome length is approximately 5 kb, with palindromic structures at the 5′ and 3′ ends.

In January 2021, the lung of a piglet were collected from a pig farm in Beijing, China. The piglet PPV was identified by an established PCR with primers specific for the VP2 gene of PPV (3). Subsequently, the PPV-positive sample was diluted into serum-free α-minimum essential medium (α-MEM) and then centrifuged at 10,000 × *g* for 10 min at 4°C. The supernatants were collected and filtered through a syringe filter with a 0.22-μm pore size. Then, the supernatant was mixed with α-MEM containing 2% fetal bovine serum (FBS) and was inoculated onto monolayers of ST cells (American Type Culture Collection [ATCC]). The third passage of the supernatant was used for DNA preparation, Viral DNA was extracted with the viral nucleic acid extraction kit II (Geneaid, Taiwan) according to the manufacturer’s instructions. Five pairs of primers were designed on the basis of conserved regions of the full-length sequence of PPV strain HN-2011 (GenBank accession number JX992846.1) (Table 1) to amplify five DNA fragments. The PCR products were gel purified and cloned into the pEASY-Blunt vector (TransGen Biotech, Beijing, China). Three or four clones from each PCR product were Sanger sequenced by Sangon Biotech (Shanghai, China). Finally, the data were assembled and annotated by Lasergene v7.0 with default parameters. A nearly complete PPV genome sequence was obtained, and the PPV strain was named BJ2. The PPV BJ2 open reading frames (ORFs) were annotated in SeqBuilder based on a manually curated PPV database retrieved from GenBank. The length of the genome is 4,474 bp, with a G+C content of 38%, and the coding region is 4,258 nucleotides. The genome contains two ORFs (ORF1 and ORF2). ORF1 encodes the nonstructural proteins NS1 (1,989 bp [662 amino acids]), NS2 (486 bp [161 amino acids]), and NS3 (324 bp [107 amino acids]), and ORF2 encodes the structural protein VP1 (2,190 bp [729 amino acids]) and VP2 (1,740 bp [579 amino acids]).

**TABLE 1:** Primer sequences

Primer name	Sequence (5′ to 3′)	Amplicon length (bp)
PPV-VP2-F	TGGTCTCCTTCTGTGGTAGG	445
PPV-VP2-R	CAGAATCAGCAACCTCAC	
PPV-F1	AGACTGCACTTCGCTCCAGAGACA	1,316
PPV-R1	TAGCAACCAACATTACCAACCAAG	
PPV-F2	GACTACATTAAAGTCTGCCATGC	1,279
PPV-R2	TCTGGTTGTTGAGATGTAGTTGGT	
PPV-F3	CGACGAAGCCTACGACAAATACAT	687
PPV-R3	CACACTCCCCATGCGTTAG	
PPV-F4	CACACGCATCAAGACTCATA	1,045
PPV-R4	TGATTAAATTGTTGCTTTGGAGC	
PPV-F5	CTAAAATTAACTCACTCATGGCA	1,209
PPV-R5	GCTTGTTTCTTTTTAAAATTTTA	

The MegAlign software tool was used to determine sequence identity. Sequence alignment analysis of the genome of BJ2 shows 95.2% to 99.4% nucleotide sequence identity with respect to previously reported PPV strains. The BJ2 strain has the greatest nucleotide identity (99.3%) with the Kresse strain (GenBank accession number U44978.1) and the Challenge strain (GenBank accession number AY684866.1) and the lowest nucleotide identity (96.4%) with the attenuated strain NADL-2 (GenBank accession number NC_001718.1). Previous studies showed that 6 major amino acid sites in the amino acid sequence of VP2 are the main factors affecting the tissue tropism and pathogenicity of PPV (4). The amino acid sequence of the VP2 protein showed 2 amino acid differences (T414A and P436T) between BJ2 and the 27a strain. There were 3 amino acid differences (E228Q, T414A, and Q419E) between BJ2 and the Kresse strain. There was only 1 amino acid substitution (Q228E) in the cluster 27a strain. These data will provide support for PPV epidemiological analyses and development of novel vaccines.

### Data availability.

The nearly complete genome sequence of PPV strain BJ2 has been deposited in GenBank under the accession number OP235938, with the SRA accession number SRR24166688.
